# Did the COVID-19 pandemic change the willingness to pay for an early warning system for infectious diseases in Europe?

**DOI:** 10.1007/s10198-021-01353-6

**Published:** 2021-07-20

**Authors:** Sebastian Himmler, Job van Exel, Werner Brouwer

**Affiliations:** 1 Erasmus School of Health Policy and Management (ESHPM), Erasmus University Rotterdam, Burgemeester Oudlaan 50, P.O. Box 1738, 3000 DR Rotterdam, The Netherlands; 2 Erasmus Centre for Health Economics Rotterdam (EsCHER), Erasmus University Rotterdam, Rotterdam, The Netherlands; 3 Erasmus School of Economics, Erasmus University Rotterdam, Rotterdam, The Netherlands

**Keywords:** Infectious disease outbreaks, COVID-19, Early warning system, Willingness to pay, Multi-country study, I18, H41

## Abstract

The COVID-19 pandemic highlights the need for effective infectious disease outbreak prevention. This could entail installing an integrated, international early warning system, aiming to contain and mitigate infectious diseases outbreaks. The amount of resources governments should spend on such preventive measures can be informed by the value citizens attach to such a system. This was already recognized in 2018, when a contingent valuation willingness to pay (WTP) experiment was fielded, eliciting the WTP for such a system in six European countries. We replicated that experiment in the spring of 2020 to test whether and how WTP had changed during an actual pandemic (COVID-19), taking into account differences in infection rates and stringency of measures by government between countries. Overall, we found significant increases in WTP between the two time points, with mean WTP for an early warning system increasing by about 50% (median 30%), from around €20 to €30 per month. However, there were marked differences between countries and subpopulations, and changes were only partially explained by COVID-19 burden. We discuss possible explanations for and implication of our findings.

**Supplementary Information:**

The online version contains supplementary material available at 10.1007/s10198-021-01353-6.

## Introduction

The current COVID-19 crisis and previous infectious disease outbreaks show that uncontrolled pandemics can have disastrous global consequences [1, 2], with recent estimates putting the global price tag of COVID-19 in terms of economic and disease consequences at 8–16 trillion dollar [3]. At the same time, the likelihood of the occurrence of pandemics, as well as the magnitude of their impact in terms of disease and economic burden, can be lowered drastically if appropriate measures are taken [4]. Pandemic prevention could, for example, consist of reducing the likelihood of zoonosis outbreaks themselves in different ways. It was estimated that a global strategy, involving measures like limiting deforestation and wildlife trade, as well as implementing early detection and control measures, would require yearly investments of over 20 billion dollars, but could be highly cost-effective [3]. Aiming to prevent and control zoonosis outbreaks early on, however, is only one, although important, piece of the puzzle of prevention of and preparedness for future pandemics [5]. Governments around the globe, independently, or on a supranational level, must ask themselves how to prepare for, or prevent, a next pandemic or similar health crisis. This also involves choices regarding how much funds can or should be invested in pandemic prevention measures, not knowing when and if such an event will occur again. As was pointed out before [6], (welfare) economic tools can assist “in the process of building preparedness for similar future events”. Next to calculations like those presented by Dobson et al. [3], information on society’s willingness to pay for pandemic prevention measures can provide useful information in this context.

This was recognized also before the COVID-19 outbreak. Himmler et al. [7] attempted to estimate the willingness for improvements in health safety provided by an international, integrated early warning system for identifying, containing, and mitigating large infectious disease outbreaks. Using a willingness to pay (WTP) experiment with samples from six European countries, they found a mean monthly WTP of €21.80 (median €10.00) per household for such a system, with large differences across countries (from € 8.89 in Hungary to €27.32 in Italy). The data for this study were collected in March 2018, 2 years before the COVID-19 outbreak, using hypothetical scenarios.

The current COVID-19 crisis provided the opportunity to test whether this willingness to pay would change now that a pandemic is reality rather than only a hypothetical scenario. Hence, we replicated the study by Himmler and colleagues in the spring of 2020, at a time when COVID-19 cases were increasing exponentially, economic consequences of the pandemic became clearer, and strict governmental measures were already imposed across Europe. This replication entailed fielding the same survey, using the same sampling approach, and same procedures to estimate and analyse WTP, to ensure maximum comparability between the two studies.

While one might expect the perceived value of such a warning system for infectious diseases to increase during a pandemic, as its usefulness may be more apparent and individuals’ preferences more informed, we aim to confirm this and explain any differences across the two time points by re-running the same models and comparing results. We also want to investigate whether differences are related to the impact of COVID-19, in terms of cases per 100,000 population, and the stringency of governmental measures at the time of sampling. In addition, by replicating different WTP scenarios in a new context (the pandemic), this study addresses common methodological questions regarding stated preference studies in general and contingent valuation WTP studies in particular, namely their sensitivity to scope and context [8]. This may provide further insights into the validity of estimates obtained through such studies and, hence, their policy relevance. While neither of the two experiments may necessarily elicit the “true” WTP, the unique setup allows us to at least attempt a more nuanced interpretation of the WTP data, which ultimately may also inform public investments into pandemic prevention.

## Methods

### Survey and willingness to pay scenarios

In the spring of 2020, we re-fielded a survey including a willingness to pay experiment, which was initially administered in 2018 to samples from the UK, Denmark, Germany, Hungary, Italy, and The Netherlands [7]. The same online panel provider was used (Dynata) to obtain samples of 500 individuals from each of these countries (as in the 2018 survey). We aimed for the same number of respondents as in the 2018 survey to ease comparability of WTP estimates across the two data collections. Using quota sampling, the country samples were aimed to be representative in terms of age and gender for the working age population (aged 65 or younger). The 2020 survey additionally included a sample of 500 individuals from northern Italy (defined as the regions north of Lazio and Umbria), where COVID-19 cases, mortality and lockdown measures were most severe at the time of sampling.

**Fig. 1:**
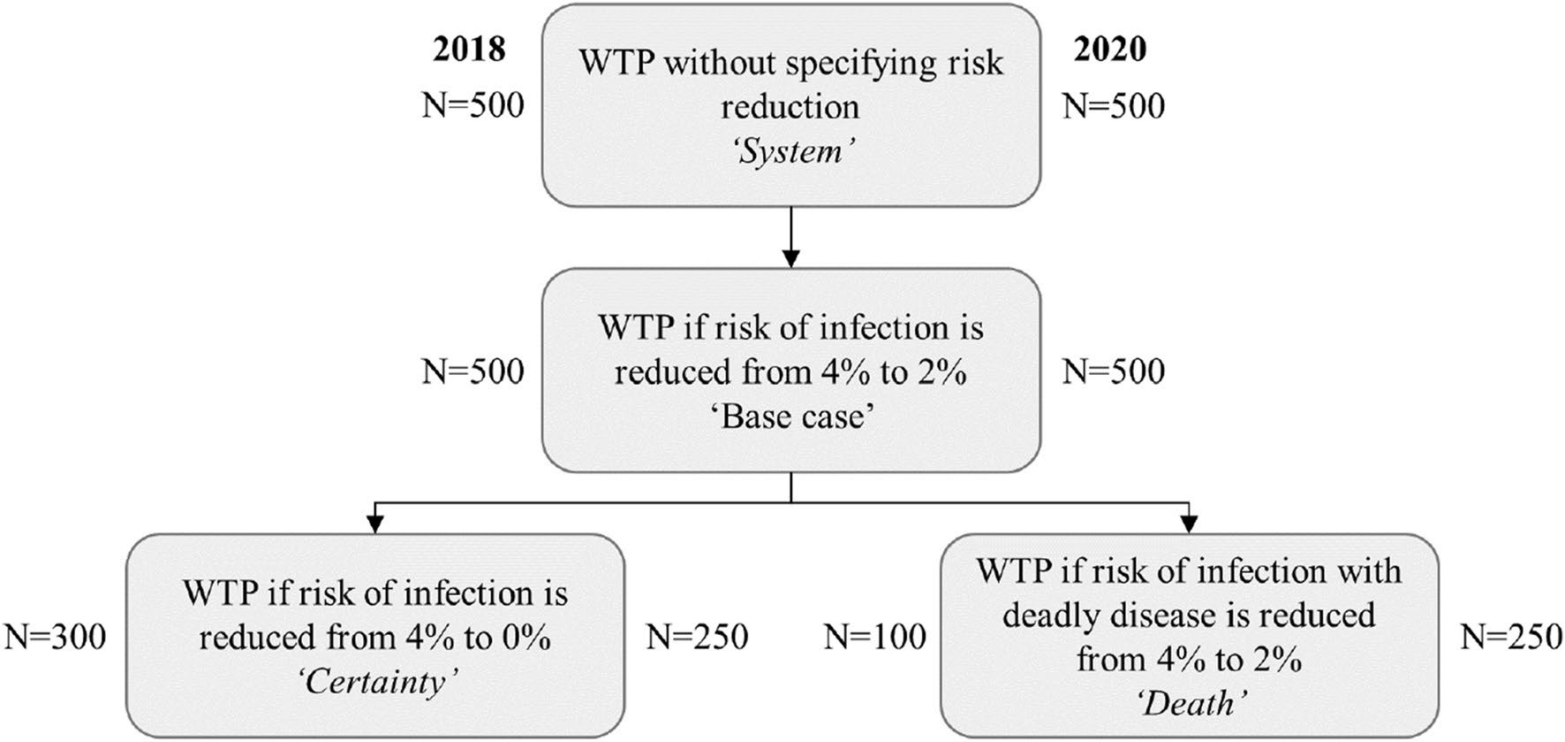
Willingness-to-pay scenarios and target samples per country for 2018 and 2020 survey. *Note.* The 2018 survey included additional scenarios not shown here. The target sample for Italy in 2020 was 1000, with 500 from northern Italy (north of Lazio and Umbria)

The contingent valuation procedure consisted of a two-step payment scale approach followed by an open-ended question to elicit the maximum willingness to pay for an integrated, international early warning system for infectious diseases. The original survey consisted of eight scenarios specifying different levels of risk reduction and (health) consequences of an outbreak; respondents all completed two basic scenarios first and were randomly assigned two of the six remaining scenarios. The 2020 survey only included the four most realistic scenarios for the current context. The flow of the WTP scenarios in the 2020 survey and the corresponding per country target samples available for analysis across the two timepoints are shown in Fig. 1. Each respondent completed three scenarios. All respondents first completed the ‘System’ and ‘Base case’ scenarios (names not shown to respondents) and were then randomized to either the ‘Certainty’ or the ‘Death’ scenario.

In the ‘System’ scenario, it was outlined to respondents that establishing and maintaining an international integrated warning system aimed at containing and mitigating infectious disease and food-borne outbreaks, like Ebola, SARS, bird flu and salmonella (COVID-19 was added to the 2020 survey), is costly. Respondents were then asked to assume that the funding would take place through national taxation via monthly instalments starting immediately and were then asked how much they would be willing to pay per month for having this international, integrated warning system. In the ‘Base case’ scenario, a 4% risk of becoming infected with a virus within the next three months was specified. If infected, health would reduce from a good to a bad health state for the duration of 1 year, which were described using EQ-5D-5L profiles corresponding to utility values of 0.887 and 0.574 (using the UK tariff from Devlin et al. [9] for all countries). Respondents were then asked to imagine that the risk to become infected can be reduced from 4 to 2% through the early warning system and subsequently had to state their willingness to pay analogous to the previous scenario. In the ‘Certainty scenario’, the risk reduction was specified to be from 4 to 0%. In the ‘Death’ scenario, the risk and the reduction were the same as in the ‘Base case’ scenario, but the consequence of an infection would be immediate death instead of a health deterioration for the duration of 1 year. Before each of the risk scenarios, respondents were made familiar with the concept of risk and probability using visual aids, similar to Bobinac et al. [10]. More details about the structure of the survey, the design of the WTP exercise, and type of survey administration and data collection can be found in the preceding study [7].

### Timing of data collection

**Fig. 2:**
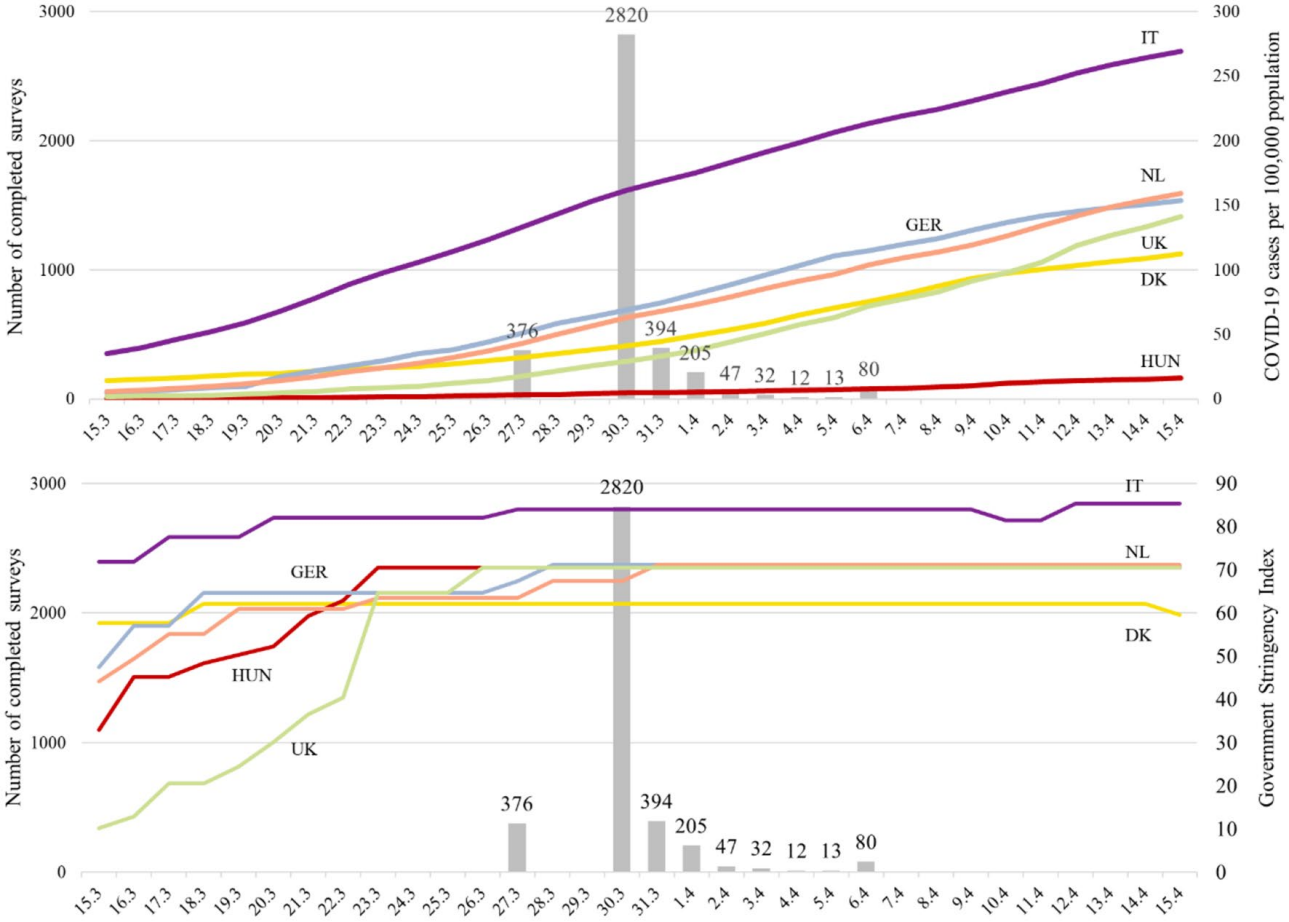
Timing of survey responses in 2020, COVID-19 cases and Government Stringency Index of measures. *Note.* Case data from ECDC [12]. Stringency index from Oxford COVID-19 Government Response Tracker [13]

In addition to the available data of 3140 observations from the 2018 survey, we were able to collect WTP responses from 3979 individuals in March/April 2020 of whom 650 also participated in the 2018 survey. Figure 2 shows the timeline of the data collection in relation to the prevalence of COVID-19 cases and the timing of restrictive policy measures in each of the included countries [11, 12]. Most of the sample was collected in the last weekend of March 2020. This was at a time when the number of cases was increasing rapidly in all included countries and restrictive policy measures, with a significant impact on peoples’ lives and daily activities, had been in place for a couple of weeks, with Hungary as the exception for both. The prevalence of COVID-19 in that period was consistently two to three times higher in Italy compared to Germany, the Netherlands, the UK and Denmark, which all experienced a similar trajectory. Throughout the sampling period, the confirmed COVID-19 cases remained at a low level in Hungary. These considerable differences between countries need to be kept in mind when interpreting the results of our analysis.

### Data analysis

Before analysing the WTP data, several steps were undertaken to facilitate a valid comparison across countries and timepoints, taking the results presented in Himmler et al. [7] as a reference. First, income and WTP values from the UK, Denmark and Hungary were converted to Euro values using the average exchange rates from March 2018 and 2020, respectively. Second, using the same criteria as in the previous study, protest answers (defined as zero response justified by warning system being a government task), and outliers (defined as a monthly WTP larger than 5% of monthly household income, which was deemed an unrealistic WTP) were identified in each of the four scenarios and excluded from the WTP analysis of the respective scenario. Third, using the country-specific consumer price indices for March 2020 from Eurostat, 2020 income and WTP values were deflated to 2018 values [14]. Fourth, country-specific monetary values were purchasing power adjusted using the latest available purchasing power parities from 2018 and the European 27 countries index as a base [15]. All monetary values reported in this study therefore represent PPP adjusted values in 2018 prices. To facilitate the comparison of regression results across the two time points when pooling country level data together, we weighted the 2020 WTP scenario observations according to the country composition in 2018. Although all country samples at both time points initially consisted of roughly 500 respondents, this was necessary as the data cleaning (protest answers and outliers) lead to unbalanced samples at both time points. The additional sample of 500 respondents from Northern Italy was omitted from these pooled regressions on the representative samples.

After these steps, mean and median willingness to pay were calculated for each scenario for all countries, the repeated sample (of respondents who participated in 2018 and 2020), and the total sample. To check whether a change in WTP would be related to changes in WTP (or ability to pay) for an everyday product, we calculated the difference in WTP between the two time points for a pair of shoes, the included warm-up WTP exercise. To facilitate a comparison, the shoe WTP values were rescaled to the mean WTP in the ‘System’ scenario in 2018.

To test if the changes in WTP across the timepoints were significantly different and not a result of differences in the samples between the two time points, we ran ordinary least squares (OLS) models in the following form, pooling the data from 2018 and 2020:

1 $${\text{WTP}}_{{{\text{isc}}}} = \alpha_{{{\text{sc}}}} + \beta_{{{\text{sc}}}} *y2020 + \gamma_{{{\text{sc}}}} *{\text{SES}}_{{{\text{isc}}}} + \varepsilon_{{{\text{isc}}}}$$

Willingness to pay values for the four scenarios $s$ and the samples $c$ (countries and combined sample) were regressed on the year indicator $y2020$, controlling for the vector $SES$ which contains the following variables: log of monthly household income, age, age-squared, gender, level of education, marital status, and employment status. Only the resulting $\beta$ parameters, which indicate the change in WTP due to the COVID-19 outbreak, will be reported. Standard errors were clustered on country level for the combined sample regression. A similar fixed effects regression, excluding the time invariant covariates, was run for the sub-sample of individuals, which were observed at both time points to account for time invariant unobservables.

Himmler et al. [7] conducted linear regression analysis to examine whether factors influencing WTP were in line with theoretical considerations, as well as previous empirical findings of WTP determinants. To test if there were meaningful shifts in the importance of these determinants between 2018 and 2020 samples, and whether these could be linked to the COVID-19 outbreak and its consequences, we repeated the analysis for both timepoints. WTP values from all four scenarios (Fig. 1) and countries were combined, increasing the number of observations and therefor the statistical power to detect significant changes.

WTP was modelled as a function of the same vector $SES$ as in Eq. (1); health status $HS$, as measured using the sum score of the EQ-5D-5L; the level of awareness of outbreaks $aware$; whether individuals or a family member have been exposed to an infectious disease outbreak before or not ($exposure$); and the health-risk attitude of respondents, which was assessed using the sum score of the six-item version of the health-risk attitude scale ($HRAS$) and included as quartile indicators [16]. The awareness variable, which was originally a sum score of 12 Likert-scale questions, was split into three sub-scores to provide more nuance: personal risk perception and behaviour, societal consequences of outbreaks, and risk and response. For the full questions, see Appendix Fig. A1.

It is important to note that the (statistical) comparison of regression coefficients from two independent samples is inherently difficult, even if the data generating process is the same and the samples should be comparable. Consistent inference on the parameters across the 2018 and 2020 samples was facilitated through Stata’s ‘suest’ command [17]. This command provides estimates for seemingly unrelated regressions using a joint variance–covariance matrix of all parameters. This allowed us to compute t-tests comparing coefficient estimates across 2018 and 2020 samples. Standard errors in the regressions were clustered on individual level to account for the dependence of WTP responses within an individual.

A significance level of 10% was used throughout the analysis. The statistical analysis of the data was performed using Stata 16.0 (Stata Corp. 2019. Stata Statistical Software: Release 16. College Station, TX: Stata Corp LP).

## Results

### Sample characteristics

Sample characteristics and country composition are presented in Table 1. While observations were otherwise equally distributed across countries, we obtained a larger sample for Italy in 2020, with 394 respondents specifically from north Italy. Information on the response rate and completion rate was not provided by the sampling agency. There were no considerable changes in overall respondent characteristics between the two sampling periods except an increase of the share of dependent employed individuals from 54 to 58% and an increase in monthly household income by 5.4% (after adjusting for inflation and purchasing power). Important to note is that the level of income was lower in the 2020 sample for Hungary (Appendix Table A1 contains country level means). The sub-sample of individuals, who were observed at both timepoints had a significant lower level of income and was significantly older than the full samples in 2018 and 2020. Furthermore, the repeated sample has been previously exposed to infectious diseases to a lesser degree (Appendix Table A2). These differences imply that the individuals who participated twice in the survey, represent a specific selection of individuals. In this hereafter called ‘repeated sample’, respondents from the UK, Germany and Italy are furthermore overrepresented, as less individuals who already participated in 2018 could be sampled from Denmark and the Netherlands. Appendix Table A4 shows the dataset conditioning for the different parts of the analysis.

**Table 1:** Characteristics of full sample and repeated sub-sample across timepoints

	Full sample		Repeated sample	
	2018	2020	2018	2020
Monthly income in €^a^	2917 (3765)	3052 (4969)	2571* (2038)	2726* (4564)
Age	42.2 (14.0)	42.7 (13.1)	43.8* (12.2)	45.8* (12.1)
Female	0.51	0.51	0.50	0.50
No finished sec. education	0.03	0.03	0.03	0.02*
Finished high school	0.57	0.57	0.58	0.58
Tertiary education	0.40	0.40	0.39	0.40
Married	0.58	0.57	0.56	0.59
Employed	0.54	0.58	0.58*	0.60
Self-employed	0.10	0.11	0.12*	0.11
Unemployed	0.06	0.08	0.08*	0.07
Homemaker	0.07	0.06	0.07	0.07
Student	0.10	0.06	0.05*	0.04*
Retired	0.09	0.08	0.07*	0.08
Unable to work	0.04	0.04	0.03	0.03
*Country*				
UK	0.18	0.16	0.19	0.19
DK	0.16	0.13	0.10	0.10
GER	0.17	0.16	0.19	0.19
HUN	0.16	0.13	0.16	0.16
IT	0.17	0.17^b^	0.25	0.15^b^
		0.10^c^		0.10^c^
NL	0.17	0.16	0.11	0.11
Observations	3,140	3,979	650	650

*Note.* ^a^In 2018 PPP. Income information was available for 2772 and 3608 respondents in full sample and 578 and 584 respondents in repeated sample. ^b^South and ^c^North Italy. *p < 0.10 in independent *t*-tests comparing repeated to full sample in the respective year

### Changes in awareness, exposure, health-risk attitude, health, and well-being

To aid in interpreting the WTP results, we will first summarise some descriptive evidence on changes in contextual factors like awareness of outbreaks, health risk attitude, past exposure, and health and well-being between the March 2018 and March 2020 samples. More detailed descriptions of these factors and/or the corresponding results are provided in Appendix 1.

Overall, the awareness or perceptions of risks and consequences of infectious disease outbreaks increased (Fig. A1). People feel more at risk compared to others, would be more willing to take precautionary measures advised by authorities, are more concerned about infectious diseases compared to other diseases, and are informing themselves about outbreaks more often. They are more aware of the damage such outbreaks can have on health, social life, and the economy, while agreeing to a much higher degree that outbreaks are a major public health concern (65–81%). Interestingly, the share of individuals, who think that the risk of outbreaks cannot be lowered by taking precautionary measures, remained almost the same (7–6%). A striking observation is that even during the COVID-19 pandemic, 45% of respondents agreed with the statement that outbreaks originate in other countries and it would be their responsibility to deal with them (44% in 2018), dismissing the need for an international response.

The sample of March 2020 was, in general, slightly more health-risk averse with a mean HRAS score (range 6–42) of 30.1 (SD 5.8) compared to the 2018 sample (28.8, SD 5.7). This shift can largely be explained by respondents agreeing to a greater extend with the statement “To enjoy good health now and in the future, I am prepared to forego a lot of things” (49% to 62%) (Fig. A2). The relative increases in awareness and health risk aversion between 2018 and 2020 were similar across all countries (Appendix Table A3). The highest levels thereof were observed for Italy for both time points.

The share of individuals reporting that they themselves or a family member have been exposed to an emerging infectious disease or foodborne outbreak in the past decreased from 19 to 16% in the total sample. In Italy, this share increased from 13 to 16% and 18% in north and south Italy, respectively. The large differences in self-reported exposure between countries (Appendix Table A3) may partly be a result of a different interpretation of the question (in 2018 the share varied from 10% in Denmark to 62% in Hungary). Similarly, observed decreases in the rate between the two timepoints could reflect more accurate responses in 2020, as respondents were likely more knowledgeable about the subject area due to the COVID-19 outbreak. In terms of the impact the COVID-19 outbreak on self-reported health, life satisfaction and capability well-being, we did not observe any meaningful changes between the 2018 and 2020 sample (Appendix Fig. A3).

### Willingness to pay across countries and timepoints

Of the total of 20,606 WTP values across the four scenarios, 1104 were classified as outliers, and 1643 as protest answers (Appendix Table A5 provides scenario level information). Dropping these observations lead to a WTP analysis sample of 17,859 observations. The share of protest answers and zero WTP responses were in general lower in 2020 compared to the 2018 sample, apart from the ‘Death’ scenario. The largest drop in the share of protest answers was observed for Hungary (e.g., from 17 to 7% for the ‘System’ scenario. The share of WTP values classified as outliers on the other hand, increased for almost all scenarios and countries.

**Fig. 3:**
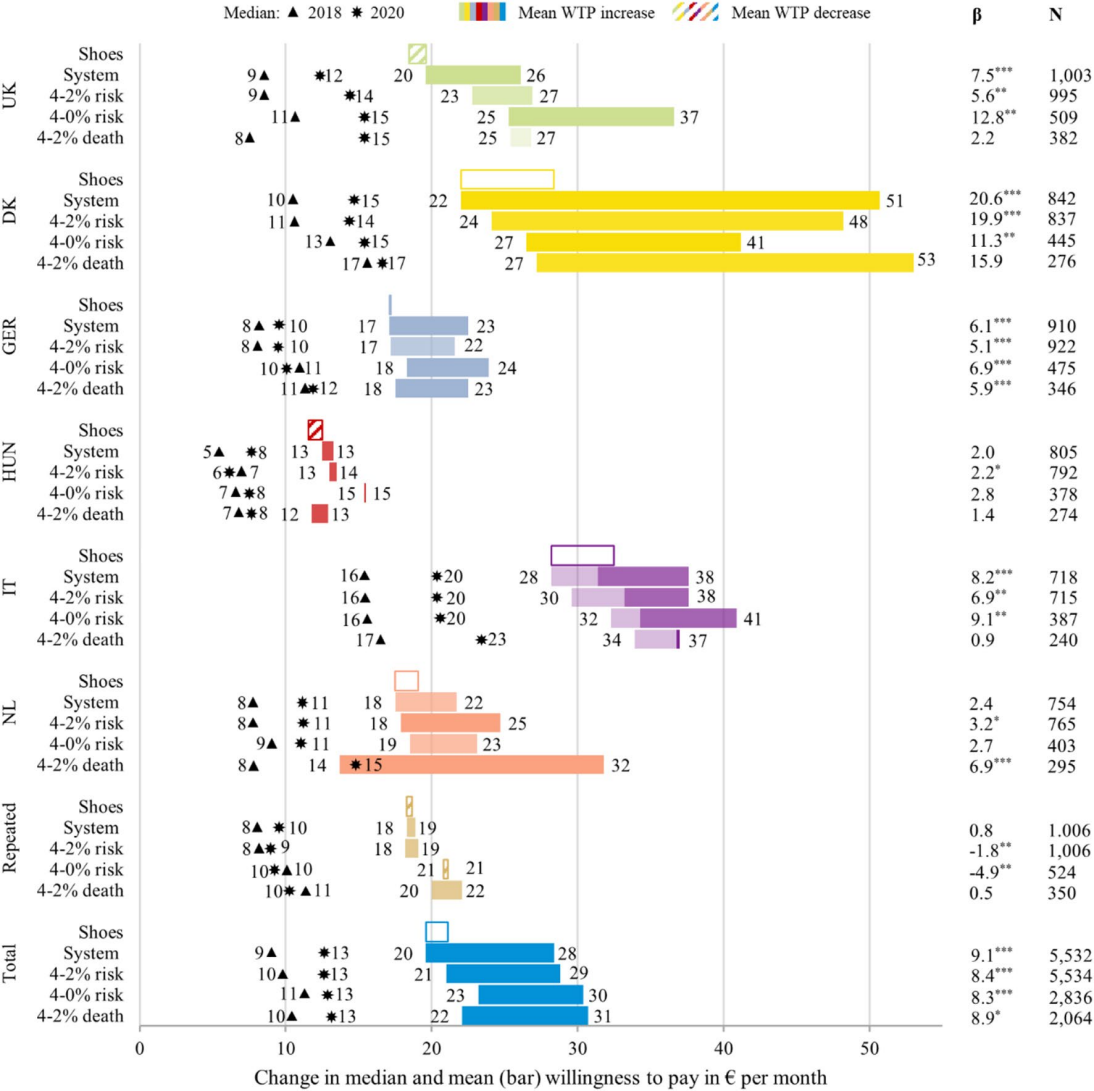
Changes in willingness to pay for an early warning system across scenarios, countries and timepoints. *Note.* WTP in 2018 PPP. Changes in mean WTP from 2018 to 2020 represented as bars. WTP for shoes as reference and rescaled to ‘System’ 2018 values. Deep color bars for Italy represent additional WTP in northern Italy compared to southern Italy and 2018. Total sample values weighted to maintain same country composition on aggregate. β parameters represent coefficients of the y2020 dummy variable from regressions on the pooled sample, controlling for log of income, age, gender, education, and marital and employment status (Eq. 1). N is the number of observations in the respective regressions. *p < 0.10 **p < 0.05 ***p < 0.01

Figure 3 presents mean and median WTP for an early warning system for infectious diseases across scenarios and countries, comparing 2018 values to the values obtained during the COVID-19 outbreak in 2020. The figure also includes the scenario and country level estimates of β, the timepoint dummy from the pooled regression analysis (Eq. 1). There is large variation in WTP values across countries, scenarios and timepoints. Important to note is that the country specific WTP values were rather stable across the four scenarios, despite the differences imposed in the scenario description (Fig. 1). The total mean WTP increased by between 30 and 40%, depending on the scenario, corresponding to an additional monthly contribution of 7€–9€ (baselines values were 20€, 21€, 23€ and €22 for the four scenarios). The total median monthly WTP increased by between €1.6–€3.6 (15–40% increase). The total variation in elicited WTP values more than doubled in each of the four scenarios. In 2018, the variation in WTP in the ‘System’ scenario was 28.6, while in 2020 the standard deviation was 71.2.

The largest increases across all scenarios where found for Denmark. There, WTP in the ‘System’ scenario almost doubled, even after accounting for differences in socio-economic characteristics (baseline 2018 value of €22, β-coefficient €20.6). Besides for the ‘Certainty’ scenario in the UK, moderate increases in monthly WTP of up to €10 were found in the remaining countries. The WTP was lowest in Hungary, with values remaining almost stable across timepoints (maximum monthly WTP increase of €2.8 and not significant). There was a larger increase in monthly WTP in northern Italy (up to €9.1) compared to the south, with the Italian sample reporting the highest levels of WTP in 2018. Interestingly, WTP was stable or even decreased in the subset of observations, which were observed twice. Results from the reference point included, WTP for a pair of shoes (rescaled to mean of the ‘System’ results for 2018), indicated that willingness and ability to pay, in general, slightly increased across the two timepoints, except for Hungary and the repeated sample.

As COVID-19 cases and governmental measures increased over the period of data collection (Fig. 2), whether certain sub-samples were collected particularly early on or later may have impacted WTP. However, we found no worrisome pattern in our data.

**Fig. 4:**
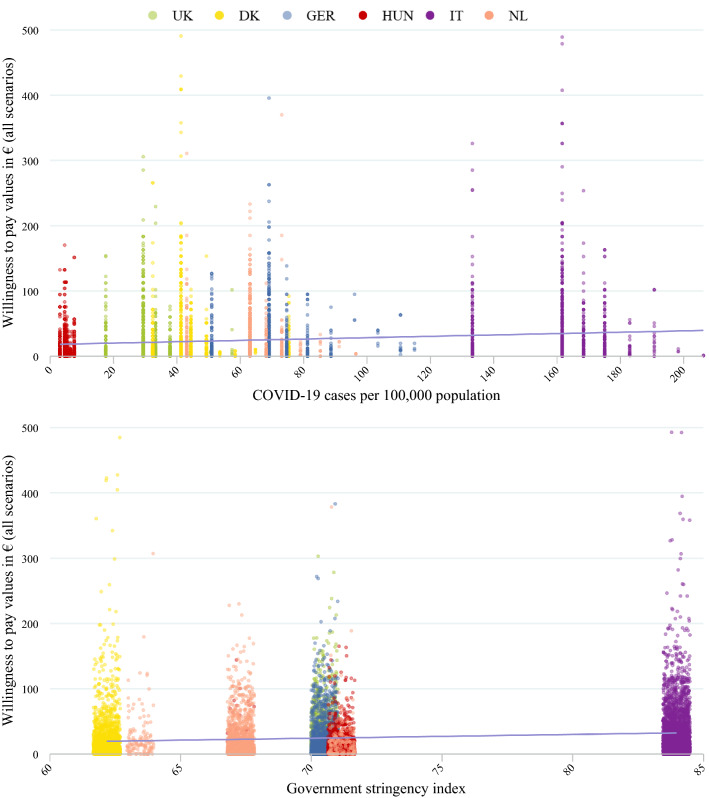
Willingness to pay during COVID-19 outbreak in relation to number of cases and measures. *Note.* Case data from ECDC [12]. Stringency index from Oxford COVID-19 Government Response Tracker [13]. Horizontal line represents linear fit. Random variation added to GSI (jitter)

Figure 4 plots willingness to pay values for the 2020 sample against the country aggregate number of COVID-19 cases and the government stringency index. There seems to be a positive relationship between number of cases and the WTP for an early warning system. Interestingly, the occurrence of extreme values seems to decrease over time (higher number of cases equals later timepoint as number of cases was consistently increasing over the sampling period) within most countries. A positive relationship was also observed for WTP and the government stringency index, although the variation in the strictness of government measures was much smaller (Fig. 2).

### Determinants of willingness to pay

Table 2 presents results of the seemingly unrelated estimation procedure, allowing the comparison of coefficients of WTP determinants across 2018 and 2020 sample. A structural difference in the overall associations was confirmed by a Chow-test, which rejected the null hypothesis of equality of coefficients in the 2018 and 2020 samples (chi-squared: 56.37, *P* < 0.01). The included variables explained a larger share of the variance in WTP in the 2020 regression. While the directions of associations with WTP remained stable for some variables (log-income, being female, tertiary educated, or self-employed, and past exposure), the estimated coefficients switched sign for variables like being married or unemployed. Besides these changes, large and significant differences in coefficient size were found for log-income, self-employment, being in the highest health-risk aversion quartile. Personal risk perception and behaviour also played a larger role for the 2020 WTP values. That coefficient estimates generally increased may partly be explained by the larger WTP values and the larger variation in WTP values found in 2020 compared to 2018 (standard deviations in all four scenarios doubled).

**Table 2:** Determinants of willingness to pay across time points

	2018		2020		*P* Difference
*Socio-economics status*					
Log income	9.41***	(1.01)	33.57***	(3.70)	< 0.001
Age (Δ5 years)	– 5.38***	(1.39)	– 2.87	(2.71)	0.399
Age-squared	0.18**	(0.08)	– 0.03	(0.15)	0.220
Female	-3.55***	(1.03)	– 2.78*	(1.67)	0.682
Tertiary education	1.21	(1.10)	4.23***	(1.48)	0.099
Married	2.17**	(1.03)	– 15.87***	(3.12)	< 0.001
Self-employed	2.06	(2.09)	38.95***	(7.65)	< 0.001
Not employed	– 2.25**	(1.14)	8.37***	(2.19)	< 0.001
EQ-5D-5L sum score (Δ5 points)	– 0.89***	(0.22)	– 0.01	(0.31)	0.019
*Awareness of outbreaks*					
Personal risk perception (Δ5 points)	6.02***	(0.82)	13.16***	(1.95)	0.007
Societal consequences (Δ5 points)	– 2.14***	(0.80)	– 1.52	(2.12)	0.784
Risk and response (Δ5 points)	– 2.07	(1.29)	– 17.31***	(3.90)	< 0.001
Past exposure	4.20***	(1.28)	2.37	(1.95)	0.432
*Health risk attitude*					
HRAS-SF Q2	0.12	(1.33)	– 2.28	(2.40)	0.382
HRAS-SF Q3	– 0.40	(1.33)	– 1.27	(2.12)	0.729
HRAS-SF Q4	4.88***	(1.56)	12.18***	(2.46)	0.011
Observations	6611		8442		
Adjusted R-squared	0.190		0.278		
Chow test statistics	56.37***	*P* < *0.01*			

*Note.* WTP values from all four scenarios as dependent variable. Standard errors were clustered on individual level and are presented in parentheses. Northern Italy subsample from 2020 excluded. Country dummies and constant omitted from the table. Regression is weighted by 2018 country sample sizes. *p < 0.10, **p < 0.05, ***p < 0.01

Appendix Table A6 presents the results for the subsample of repeated observations. As differences in WTP between the two timepoints were considerably less pronounced in this sample, changes in the importance of determinants occurred less frequently. The Chow test further confirms no structural change in overall coefficients between the two timepoints (chi-squared: 19.21, *P* = 0.57). In general, the variables followed a similar pattern compared to the full sample. A notable exception is that the coefficient of self-employment did not increase. No structural change in coefficients estimates was also found in the subsamples of respondents from Italy based on the Chow test (chi-squared: 22.53, *P* = 0.13).^1^ Interestingly, the coefficient of past exposure decreased (Appendix Table A7).

## Discussion

### Summary of WTP results

During the onset of the COVID-19 crisis in Europe, we repeated an experiment from 2018 by Himmler et al. which elicited the WTP for improvements in health safety provided by an international, integrated early warning system for identifying, containing and mitigating large infectious disease outbreaks. Overall, we found statistically significant increases in mean monthly WTP by about 50%, depending on the specified WTP scenario (e.g., from €20 to €28 in the ‘System’ scenario), while the corresponding medians increased by about 30% (e.g., from €9 to €13 in the ‘System’ scenario). Differences between countries were more pronounced compared to the 2018 data collection. The largest increases in WTP were observed for the UK, Denmark, and Italy. We furthermore found rather stable WTP values in a sub-sample of individuals before and during the COVID-19 outbreak. Most of these individuals did not, or only slightly changed their WTP between the two timepoints (Appendix Fig. A4).

### Possible explanations of changes and patterns in WTP

The observed *moderate* increases in WTP for an early warning system for infectious diseases elicited pre-pandemic and during the first wave of COVID-19 in Europe may be interpreted in different ways. An optimistic interpretation is that the experiments set out to elicit WTP twice for the same good: an early warning system. The fact that the resulting WTP estimates at both points in time were not considerably different could signal that the anticipated risks and consequences of pandemics influencing the WTP during the first experiment were similar to the more informed ones during the second experiment. The higher awareness of outbreaks, and the risk and consequences of their occurrence, which we observed, then lead to respondents forming reasonable and realistic increases in WTP given their ability to pay. The elicited WTP estimates in 2020 then constitute an upper bound, as the COVID-19 pandemic and its consequences likely and hopefully remain an extreme variant of an infectious disease outbreak.

A more pessimistic interpretation would be that the chosen approach does not invite respondents to reveal changing preferences, for instance due to insensitivity to scale and scope in the elicitation technique. The methods used in, as well as the framing and scope of the experiment, may then not adequately reflect changes in ‘actual WTP’ following the COVID-19 outbreak. Although our data do suggest that there is some plausible sensitivity in our results and also some patterns that represent logical deviations from the initial WTP estimates, we cannot fully disentangle or refute the optimistic and pessimistic interpretations of our findings.

In terms of the differences in changes between countries, the finding that WTP in Italy increased more than for instance in Germany, the Netherlands, and Hungary, may not be unexpected given that Italy was hit hardest by the pandemic in March/April 2020. We did not find considerable changes in WTP in Hungary, which could be related to the fact that, at the time of data collection, it was the least affected country. On an individual level, WTP values during the COVID-19 outbreak seem to be determined to a higher extent by respondent characteristics. This relates to the potential impact of such a pandemic on individuals’ lives and livelihoods, or the perceived individual (health) risks, as well as attitudes towards these risks. The most notable change in WTP determinants was observed for being self-employed. This is in line with first evidence from Germany, indicating that self-employed individuals were hit hardest by the pandemic in terms of economic consequences [18]. Gross monthly income was reduced for 59% of self-employed (vs. 15% of employed), with a median reduction of €1,500.

At the same time, we also found patterns that may be considered more unexpected. For instance, the increase in WTP in Northern Italy was small in relation to the severity of the crisis there during data collection. This contradicts the explanation that WTP is importantly influenced by the severity of the crisis. A potential explanation for this finding could be that Italians in this region were relatively dissatisfied with the COVID-19 response of their government, as well as with the assistance from the international community [19]. This could have decreased their trust in the possibility of an effective integrated international early warning system. It is also important to note that WTP was already highest in Italy in the 2018 sample, arguably leaving less room for further increases. Likewise, finding a high WTP for the early warning system in Denmark does not appear to correlate with the COVID-19 burden in that country (Fig. 2). There, it may relate both to higher incomes and the high level of trust in national public institutions and the government [20], which also prevailed during (the early phase of) the pandemic [21]. A further possible explanation for country-level WTP changes not being directly related, or at times being even reversely related to the burden of COVID-19, is that contributing to a preventive system now, actually does not help to overcome the *current* crisis. Respondents may feel that the current crisis should be given priority in terms of public expenditures, especially if the COVID-19 burden is severe. In that sense, it is good to highlight the difference between our study considering *preventative actions,* compared to curative or mitigating actions, for instance asking about the WTP for a vaccine. Again, given the setup of our study we cannot be conclusive regarding these potential influences.

Another aspect, which may have influenced WTP values elicited during the pandemic, could be that individuals anticipated an economic downturn, and the personal consequences thereof, as a result of the pandemic. Therefore, they might be less willing (or able) to pay additional taxation. However, results from the non-health-related reference point included in our survey (WTP for a pair of shoes) and the income information indicated that, on average, the ability to pay (for everyday products at least) was not yet significantly affected by the COVID-19 outbreak. Indeed, the first noticeable economic consequences of the pandemic likely occurred after our sampling period in March 2020. Also, respondents in the second data collection may have been more aware of the fact that such a system would help to avoid later losses in income. This could have resulted in an increased willingness to pay, since they were more aware of the benefits of such a system for their own economic situation.

These explanations may also have caused WTP values to be fairly stable in the subgroup of respondents who completed the survey at both moments in time. In addition, it is important to note that respondents in this subgroup had a lower income (Table 1), and had a lower level of previous exposure to infectious diseases (Table A2). The country composition in this sample also did not reflect the original sampling quotas (equal across countries) and the individuals, who were observed twice, were different compared to the samples in their respective countries (Table A2). That we found a small *decrease* in ability to pay (as measured via the WTP for shoes scenario) for the repeated sample, which is in contrast to what was found for most included country samples, further highlights that this sample represents a specific selection of individuals.

### Additional findings

We found notable shares of protest answers and zero responses. Moreover, a large share of respondents at the time were not convinced of the need of an international response during the COVID-19 outbreak. Furthermore, a significant proportion of respondents were still not aware of (or ignored) the seriousness of the societal impact of an outbreak, as well as the fact that precautionary measures could decrease the risk of outbreaks (Sec. “Changes in awareness, exposure, health-risk attitude, health, and well-being”). These individuals may therefore disapprove of the governmental measures taken and might be hesitant to take up vaccination if available [21, 22].

Finding no differences in well-being and life satisfaction between the 2018 and 2020 samples may be somewhat surprising. The fact that the survey was fielded at a time when the *full* impact of the crisis on individuals’ well-being and the economy at large was not clear to respondents (Fig. 2) may help to explain this. A study from Germany, comparing individuals from a large panel sample across April 2020 and April 2019, also did not find a change in life satisfaction due to the COVID-19 outbreak [23]. Capability well-being, as assessed by the ICECAP-A, which specifically aims to measure capabilities and opportunities, was also not lower in our second survey compared to the first, even though the COVID-19-related lockdowns imposed quite drastic limitations on individuals’ freedom and rights. It is interesting to see how such outcomes will evolve during the crisis, especially when these restrictions are imposed for longer periods of time.

### Limitations of the analysis

Similar limitations as were outlined in more detail in the first study [7] apply to the current study as well. These relate to more general limitations of stated preferences and contingent valuation approaches, such as hypothetical response bias, insensitivity to scope, and framing effects [8, 24]. These limitations are particularly important when the good under valuation is less tangible to respondents. This clearly applies here, as the early warning system for infectious diseases and its consequences are still hypothetical. Respondents therefore may have had difficulties in imagining such a system and its potential costs and benefits. Insensitivity to scope, which has been shown to exist before in the health domain using a similar setup [25], was evident in our analysis considering the WTP results for the different presented scenarios. The small difference across scenarios may also be a result of respondents anchoring their WTP on their valuation of the first presented WTP scenario (‘System’ scenario). Insensitivity to scope may also explain the relative insensitivity of observed WTP values to the changes in circumstances over time, i.e., the COVID-19 outbreak. Regarding the hypothetical nature of the experiments, the following is important to note: the COVID-19 outbreak made the pandemic scenario more *real*. However, whether that made the presented WTP scenarios (Fig. 1) more *realistic* for an average respondent, is unclear. If scenarios were not recognized as relevant for the COVID-19 situation (e.g., because not so many people will be infected or die, or because the health states were not deemed plausible in relation to COVID-19), the scenarios possibly remained as hypothetical as in the first data collection.

In terms of the comparison of the 2018 and 2020 WTP values, it needs to be acknowledged that ideally, we would have resampled the full 2018 survey population. This would have enabled us to compare the same individuals within representative country samples. Although attempted this turned out not to be possible, and hence we needed to assume that the representative samples from 2018 and 2020 did not differ too much in terms of unobserved characteristics, which would have influenced WTP. For example, the data collection during the COVID-19 outbreak on such a topic could have attracted specific populations who would sooner select into participating in a survey on this topic. On the other hand, we took several steps, to enable a (valid) comparison, like PPP adjusting, accounting for inflation, weighting sample compositions or controlling for observable characteristics in the year-dummy regressions.

A final limitation concerning our sample is that we do not have WTP information from individuals aged 65 and older, which are the ones with the highest risk of serious health consequences due to a COVID-19 infection. The sample of 65 and younger may be seen as primarily (though clearly not exclusively) affected by economic consequences. This may be a reason why we found that age and health were not significant determinants of WTP in 2020, while self-employment and unemployment were. One might hypothesize that the largest changes in WTP over time may have occurred in the risk group of individuals aged 65 and above, which were not included in our samples. This reduces the generalisability of our findings, especially in contexts where the financing of an early warning system would be based on contributions from all citizens, including those older than 65 years of age.

While not a limitation, it is important to note that our study focused on European countries and similar experiments may have led to very different WTP results in other parts of the world even after PPP adjustment. Knowledge about COVID-19 and the public’s perception of the pandemic and the associated risks, factors likely influencing WTP, vary widely across the globe [26–28]. In addition, the measures taken against COVID-19 between for example Europe and East Asia are different, thus may also translate to differences in WTP for an early warning system, as individuals would value efforts for either adopting or avoiding these measures more depending on individual values. One prominent example relate to the type of isolation used (or mandated) for mild COVID-19 patients [29, 30], where East Asian countries such as China adopted facility-based isolation with financial support and mental health counselling and lowered patients' anxiety to transmit virus to family members, yet this may not be valued in Western countries such as the UK due to privacy infringements.

## Conclusions

Repeating a European survey from 2018 eliciting the WTP for an early warning system aimed to prevent or mitigate outbreaks of infectious diseases, we found a higher WTP in 2020 as compared to 2018 in all countries except Hungary. We also observed a considerable increase in the heterogeneity in elicited values (both within and between country samples). Respondents showed some sensitivity to scope and to the context of the experiment (the COVID-19 outbreak), oftentimes in expected directions. However, the sensitivity to scope and context varied and should be interpreted with caution (see e.g., Bobinac et al. (2012)). Our results should therefore be taken to represent a range of WTP values rather than a precise estimate of some ‘true’ WTP for an early warning system. We also stress that the contingent valuation WTP method has notable limitations, especially given the abstract nature of an early warning system. Nonetheless, also in the absence of clearly better alternatives, our study aims to provide a relevant indication of the societal valuation by European citizens of such an early warning system for infectious diseases. Conducting a back-of-the-envelope calculation aggregating the median WTP values at the country level (similar to [7]),^2^ the *moderate* increases in individual WTP translate into sizable increases at a societal level. The implied yearly maximum ‘willingness to be taxed’ increased from €1.3bn to €1.9bn in the UK, or from €6.0bn to €7.8bn summed over all six countries (Appendix Table A8).

Which of these two estimates, ex-ante or during the pandemic, is considered more informative depends also on the expected context of future outbreaks, which likely will have less extreme trajectories compared to COVID-19. Moreover, our study was conducted in the early stages of the pandemic when both duration and full societal impact were still unclear. Together with further related research during the subsequent stages of the pandemic, and also information on the (cost-)effectiveness of measures to prevent and control infectious disease outbreaks, this may inform policy makers on the type and magnitude of possible investments to prevent future outbreaks or mitigate their consequences.

## Supplementary Information

Below is the link to the electronic supplementary material.

Supplementary file1 (DOCX 954 KB)

## Data Availability

Data and code used in the study can be accessed on the Open Science Framework: https://osf.io/2wtve/.

## Footnotes

1 Excluding the additional sample from northern Italy in 2020, as we did not have comparable data for this sample for 2018.

2 Multiplying the median yearly WTP in the ‘System scenario’ with the number of households per country, excluding the share of households with protest zero answers, and assuming that 50% of the remaining households are eligible for this form of taxation.

## References

[1] Bloom, D., Kuhn, M., Prettner, K.: Modern Infectious Diseases: Macroeconomic Impacts and Policy Responses. NBER Work. Pap. No. 27757 (2020). https://www.nber.org/papers/w27757

[2] Morens DM, Fauci AS (2020). Emerging pandemic diseases: How we got to COVID-19. Cell.

[3] Dobson AP, Pimm SL, Hannah L, Kaufman L, Ahumada JA, Ando AW, Bernstein A, Busch J, Daszak P, Engelmann J, Kinnaird MF, Li BV, Loch-Temzelides T, Lovejoy T, Nowak K, Roehrdanz PR, Vale MM (2020). Ecology and economics for pandemic prevention. Science.

[4] Morse SS, Mazet JAK, Woolhouse M, Parrish CR, Carroll D, Karesh WB, Zambrana-Torrelio C, Lipkin WI, Daszak P (2012). Prediction and prevention of the next pandemic zoonosis. Lancet.

[5] Osterhaus A, Mackenzie J (2020). Pandemic preparedness planning in peacetime: what is missing?. One Heal. Outlook..

[6] Chilton S, Nielsen JS, Wildman J (2020). Beyond COVID-19: How the ‘dismal science’ can prepare us for the future. Heal. Econ. (United Kingdom)..

[7] Himmler S, van Exel J, Perry-Duxbury M, Brouwer W (2020). Willingness to pay for an early warning system for infectious diseases. Eur. J. Heal. Econ..

[8] Kling CL, Phaneuf DJ, Zhao J (2012). From Exxon to BP: has some number become better than no number?. J. Econ. Perspect..

[9] Devlin NJ, Shah KK, Feng Y, Mulhern B, van Hout B (2018). Valuing health-related quality of life: An EQ-5D-5L value set for England. Heal. Econ. (United Kingdom).

[10] Bobinac A, van Exel J, Rutten FFH, Brouwer WBF (2014). The Value of a QALY: Individual Willingness to Pay for Health Gains Under Risk. Pharmacoeconomics.

[11] HIQA: Review of restrictive public policy measures to limit the spread of COVID-19 (2020). https://www.hiqa.ie/reports-and-publications/health-technology-assessment/review-restrictive-public-policy-measures

[12] ECDC: Data on the geographic distribution of COVID-19 cases worldwide (2020). https://www.ecdc.europa.eu/en/publications-data/download-todays-data-geographic-distribution-covid-19-cases-worldwide

[13] Hale, T., Webster, S., Petherick, A., Phillips, T., Beatrz, K.: Oxford COVID-19 Government Response Tracker. Blavatnik School of Government. Data use policy: Creative Commons Attribution CC BY standard (2020). https://www.bsg.ox.ac.uk/research/research-projects/covid-19-government-response-tracker

[14] Eurostat: Harmonised index of consumer prices - monthly data (2020). https://appsso.eurostat.ec.europa.eu/nui/show.do?dataset=prc_hicp_midx&lang=en

[15] Eurostat: GDP per capita in PPS. Index (EU28 = 100) (2020). https://ec.europa.eu/eurostat/tgm/table.do?tab=table&init=1&language=en&pcode=tec00114&plugin=1

[16] Huls S, van Osch S, Brouwer W, van Exel J, Stiggelbout A (2020). Psychometric evaluation of the Health-Risk Attitude Scale (HRAS-13): Assessing the reliability, dimensionality and validity in the general population and a patient population. Psychol. Heal..

[17] Weesie J (2000). Seemlingly unrelated estimation and the cluster-adjusted sandwich estimator. Stata Tech. Bull..

[18] Kritikos, V.A.S., Graeber, D.: Corona-Pandemie wird zur Krise für Selbständige. DIW aktuell. 47 (2020)

[19] European Parliament: Public opinion monitoring at a glance in the time of COVID-19 (2020). https://www.europarl.europa.eu/at-your-service/de/be-heard/eurobarometer/public-opinion-in-the-time-of-covid-19

[20] Marozzi M (2015). Measuring Trust in European Public Institutions. Soc. Indic. Res..

[21] Sabat I, Neuman-Böhme S, Varghese NE, Barros PP, Brouwer W, van Exel J, Schreyögg J, Stargardt T (2020). United but divided: Policy responses and people’s perceptions in the EU during the COVID-19 outbreak. Health Policy.

[22] Neumann-Böhme S, Varghese NE, Sabat I, Barros PP, Brouwer W, van Exel J, Schreyögg J, Stargardt T (2020). Once we have it, will we use it? A European survey on willingness to be vaccinated against COVID-19. Eur. J. Heal. Econ..

[23] Entringer, T., Kröger, H.: Einsam, aber resilient – Die Menschen haben den Lockdown besser verkraftet als vermutet. DIW aktuell. 46 (2020)

[24] List JA, Gallet CA (2001). What experimental protocol influence disparities between actual and hypothetical stated values?. Environ. Resour. Econ..

[25] Bobinac A, van Exel NJA, Rutten FFH, Brouwer WBF (2012). GET MORE, PAY MORE? An elaborate test of construct validity of willingness to pay per QALY estimates obtained through contingent valuation. J. Health Econ..

[26] Yu F, Geldsetzer P, Meierkord A, Yang J, Chen Q, Jiao L, Abou-Arraj NE, Pan A, Wang C, Bärnighausen T, Chen S (2021). Knowledge About COVID-19 Among Adults in China: Cross-sectional Online Survey. J. Med. Internet Res..

[27] Geldsetzer P (2020). Use of Rapid Online Surveys to Assess People’s Perceptions During Infectious Disease Outbreaks: A Cross-sectional Survey on COVID-19. J. Med. Internet Res..

[28] Chen S, Chen Q, Yang J, Lin L, Li L, Jiao L, Geldsetzer P, Wang C, Wilder-Smith A, Bärnighausen T (2021). Curbing the COVID-19 pandemic with facility-based isolation of mild cases: a mathematical modeling study. J. Travel Med..

[29] Chen S, Zhang Z, Yang J, Wang J, Zhai X, Bärnighausen T, Wang C (2020). Fangcang shelter hospitals: a novel concept for responding to public health emergencies. Lancet.

[30] Chia ML, Him Chau DH, Lim KS, Yang Liu CW, Tan HK, Tan YR (2021). Managing COVID-19 in a novel, rapidly deployable community isolation quarantine facility. Ann. Intern. Med..

