# Supplementary material for: Did the COVID-19 pandemic change the willingness to pay for an early warning system for infectious diseases in Europe?
Source: Eur J Health Econ. 2021 Jul 20;23(1):81–94. doi: 10.1007/s10198-021-01353-6 (PMC8294297; doi:10.1007/s10198-021-01353-6)
Supplement: Supplementary file 1 — Supplementary file1 (DOCX 954 KB) [file 10198_2021_1353_MOESM1_ESM.docx]

Did the COVID-19 pandemic change the willingness to pay for an early warning system for infectious diseases in Europe?

**Online Appendix**

Table A 1 Sample characteristics across countries and timepoints.

|  | **UK** | | **DK** | | **GER** | | **HUN** | | **IT** | **IT north** | **IT south** | **NL** | |
| --- | --- | --- | --- | --- | --- | --- | --- | --- | --- | --- | --- | --- | --- |
| **Full sample** | **2018** | **2020** | **2018** | **2020** | **2018** | **2020** | **2018** | **2020** | **2018** | **2020** | **2020** | **2018** | **2020** |
| Monthly income in €^†^ | 3,150 | 3,539 | 4,975 | 5,529 | 2,501 | 2,742 | 1,711 | 1,513 | 2,572 | 2,948 | 2,479 | 2,089 | 2,561 |
| Age | 42.1 | 42.7 | 41.0 | 43.4 | 43.1 | 44.2 | 41.8 | 42.1 | 41.7 | 42.8 | 40.6 | 43.5 | 43.4 |
| Female | 0.50 | 0.52 | 0.49 | 0.49 | 0.52 | 0.52 | 0.51 | 0.53 | 0.52 | 0.49 | 0.50 | 0.49 | 0.50 |
| No finished sec. education | 0.02 | 0.03 | 0.08 | 0.10 | 0.02 | 0.03 | 0.03 | 0.03 | 0.02 | 0.00 | 0.01 | 0.03 | 0.03 |
| Finished high school | 0.50 | 0.49 | 0.54 | 0.49 | 0.65 | 0.66 | 0.55 | 0.61 | 0.60 | 0.63 | 0.56 | 0.59 | 0.54 |
| Tertiary education | 0.48 | 0.48 | 0.38 | 0.40 | 0.33 | 0.31 | 0.42 | 0.36 | 0.39 | 0.37 | 0.43 | 0.38 | 0.43 |
| Married | 0.60 | 0.56 | 0.52 | 0.47 | 0.58 | 0.56 | 0.62 | 0.62 | 0.57 | 0.60 | 0.58 | 0.57 | 0.58 |
| Employed | 0.56 | 0.63 | 0.49 | 0.50 | 0.58 | 0.64 | 0.66 | 0.62 | 0.44 | 0.59 | 0.47 | 0.52 | 0.61 |
| Self-employed | 0.09 | 0.08 | 0.06 | 0.11 | 0.10 | 0.07 | 0.08 | 0.11 | 0.19 | 0.15 | 0.17 | 0.08 | 0.08 |
| Unemployed | 0.06 | 0.06 | 0.08 | 0.08 | 0.04 | 0.03 | 0.04 | 0.08 | 0.10 | 0.09 | 0.15 | 0.06 | 0.06 |
| Homemaker | 0.11 | 0.08 | 0.03 | 0.02 | 0.07 | 0.05 | 0.04 | 0.05 | 0.09 | 0.08 | 0.09 | 0.06 | 0.06 |
| Student | 0.06 | 0.04 | 0.17 | 0.09 | 0.08 | 0.06 | 0.07 | 0.05 | 0.10 | 0.03 | 0.08 | 0.11 | 0.06 |
| Retired | 0.08 | 0.06 | 0.13 | 0.14 | 0.12 | 0.12 | 0.10 | 0.09 | 0.08 | 0.06 | 0.03 | 0.06 | 0.03 |
| Unable to work | 0.05 | 0.05 | 0.05 | 0.06 | 0.01 | 0.03 | 0.02 | 0.02 | 0.00 | 0.00 | 0.01 | 0.11 | 0.11 |
| Observations | 553 | 635 | 514 | 527 | 522 | 638 | 504 | 532 | 523 | 394 | 669 | 524 | 584 |

*Note.* ^†^In 2018 PPP.

Table A 2 Characteristics of combined 2018 and 2020 sample and repeated sample across countries

|  | **UK** | | **DK** | | **GER** | | **HUN** | | **IT** |  | **NL** | |
| --- | --- | --- | --- | --- | --- | --- | --- | --- | --- | --- | --- | --- |
| **Repeated sample** | **Full** | **Rep.** | **Full** | **Rep.** | **Full** | **Rep.** | **Full** | **Rep.** | **Full** | **Rep.** | **Full** | **Rep.** |
| Monthly income in €^†^ | **3,360** | **3,321** | **5,257** | **3,987** | **2,633** | **2,442** | **1,608** | **1,629** | **2,625** | **2,521** | **2,343** | **2,197** |
| Age | **42.38** | **45.83** | **42.21** | **46.82** | **43.72** | **45.84** | **41.96** | **44.18** | **41.49** | **42.80** | **43.47** | **44.84** |
| Female | 0.51 | 0.51 | 0.49 | 0.37 | 0.52 | 0.58 | 0.52 | 0.52 | 0.50 | 0.47 | 0.50 | 0.54 |
| No finished sec. education | 0.03 | 0.02 | 0.09 | 0.05 | 0.03 | 0.03 | 0.03 | 0.01 | 0.01 | 0.01 | 0.03 | 0.01 |
| Finished high school | 0.50 | 0.51 | 0.51 | 0.45 | 0.65 | 0.68 | 0.58 | 0.58 | 0.59 | 0.61 | 0.57 | 0.60 |
| Tertiary education | 0.48 | 0.47 | 0.39 | 0.49 | 0.32 | 0.29 | 0.39 | 0.40 | 0.40 | 0.38 | 0.41 | 0.39 |
| Married | 0.58 | 0.60 | 0.49 | 0.47 | 0.57 | 0.53 | 0.62 | 0.64 | 0.58 | 0.57 | 0.58 | 0.59 |
| Employed | 0.60 | 0.61 | 0.50 | 0.56 | 0.61 | 0.64 | 0.64 | 0.68 | 0.49 | 0.46 | 0.56 | 0.66 |
| Self-employed | 0.08 | 0.10 | 0.08 | 0.05 | 0.08 | 0.11 | 0.09 | 0.09 | 0.17 | 0.19 | 0.08 | 0.04 |
| Unemployed | 0.06 | 0.05 | 0.08 | 0.13 | 0.04 | 0.02 | 0.06 | 0.03 | 0.12 | 0.15 | 0.06 | 0.07 |
| Homemaker | 0.09 | 0.10 | 0.02 | 0.02 | 0.06 | 0.06 | 0.04 | 0.04 | 0.09 | 0.09 | 0.06 | 0.09 |
| Student | 0.05 | 0.02 | 0.13 | 0.07 | 0.07 | 0.03 | 0.06 | 0.01 | 0.08 | 0.07 | 0.08 | 0.05 |
| Retired | 0.07 | 0.05 | 0.14 | 0.12 | 0.12 | 0.11 | 0.09 | 0.11 | 0.05 | 0.04 | 0.05 | 0.01 |
| Unable to work | 0.05 | 0.06 | 0.05 | 0.05 | 0.03 | 0.02 | 0.02 | 0.03 | 0.01 | 0.00 | 0.11 | 0.08 |
| **Awareness of outbreaks** |  |  |  |  |  |  |  |  |  |  |  |  |
| Personal risk perception | 21.70 | 21.37 | 20.86 | 20.91 | 20.84 | 20.56 | 21.07 | 20.87 | 22.76 | 22.09 | 20.45 | 20.13 |
| Societal consequences | 21.82 | 22.04 | 20.95 | 21.87 | 21.28 | 21.22 | 22.21 | 21.53 | 23.08 | 22.28 | 20.90 | 21.01 |
| Risk and response | 13.91 | 13.96 | 13.28 | 13.33 | 13.01 | 12.92 | 13.54 | 13.56 | 14.13 | 13.67 | 13.41 | 13.74 |
| Past exposure | **0.15** | **0.08** | **0.29** | **0.21** | **0.28** | **0.25** | **0.56** | **0.57** | **0.16** | **0.13** | **0.30** | **0.24** |
| HRAS-SF sum score | 29.54 | 30.55 | 28.31 | 28.15 | 29.33 | 29.43 | 29.44 | 29.58 | 30.60 | 30.20 | 29.40 | 29.06 |
| Cantrils ladder | 6.42 | 6.35 | 6.98 | 6.69 | 6.49 | 6.47 | 6.11 | 6.00 | 6.44 | 6.28 | 7.03 | 6.95 |
| SWLS | 20.82 | 20.96 | 22.08 | 21.47 | 21.79 | 21.96 | 18.88 | 18.71 | 20.65 | 20.23 | 23.06 | 22.47 |
| ICECAP sum score | 14.65 | 14.66 | 15.36 | 15.02 | 10.58 | 10.82 | 14.14 | 14.05 | 13.69 | 13.29 | 9.77 | 9.84 |
| EQ-5D-5L sum score | 22.40 | 22.83 | 22.30 | 22.10 | 22.18 | 22.15 | 23.10 | 22.60 | 22.68 | 22.50 | 22.82 | 22.79 |
| Observations | 1188 | 246 | 1041 | 130 | 1160 | 244 | 1036 | 210 | 1586 | 330 | 1108 | 140 |

*Note.* ^†^In 2018 PPP.

Table A 3 Awareness of outbreak risk and consequences, health risk attitude and past exposure across countries and timepoints.

|  | **Personal risk perception and behaviour** | | **Societal consequences of outbreaks** | | **Risk of and response to outbreak** | |  | **Health risk attitude scale** | | **Past personal or family exposure to outbreak** | |
| --- | --- | --- | --- | --- | --- | --- | --- | --- | --- | --- | --- |
|  | **2018** | **2020** | **2018** | **2020** | **2018** | **2020** |  | **2018** | **2020** | **2018** | **2020** |
| UK | 19.9 | 23.3 | 20.3 | 23.1 | 12.7 | 15.0 |  | 29.3 | 29.7 | 0.10 | 0.19 |
| DK | 19.1 | 22.5 | 19.4 | 22.5 | 12.4 | 14.1 |  | 27.2 | 29.4 | 0.33 | 0.26 |
| GER | 19.0 | 22.3 | 20.3 | 22.1 | 12.3 | 13.6 |  | 28.9 | 29.7 | 0.28 | 0.29 |
| HUN | 19.4 | 22.7 | 21.0 | 23.4 | 12.3 | 14.7 |  | 28.7 | 30.2 | 0.62 | 0.49 |
| IT – IT north | 21.0 | 23.6 | 21.3 | 24.0 | 12.9 | 14.7 |  | 30.1 | 31.0 | 0.13 | 0.16 |
| IT – IT south | 21.0 | 23.7 | 21.3 | 24.0 | 12.9 | 14.7 |  | 30.1 | 30.8 | 0.13 | 0.18 |
| NL | 18.3 | 22.4 | 19.2 | 22.4 | 12.4 | 14.3 |  | 28.8 | 29.9 | 0.31 | 0.28 |
| Repeated | 19.6 | 22.7 | 20.4 | 23.1 | 12.5 | 14.6 |  | 29.3 | 30.1 | 0.24 | 0.22 |
| **Total** | 19.5 | 22.9 | 20.2 | 23.0 | 12.5 | 14.4 |  | 28.8 | 30.1 | 0.29 | 0.26 |

*Note.*

**
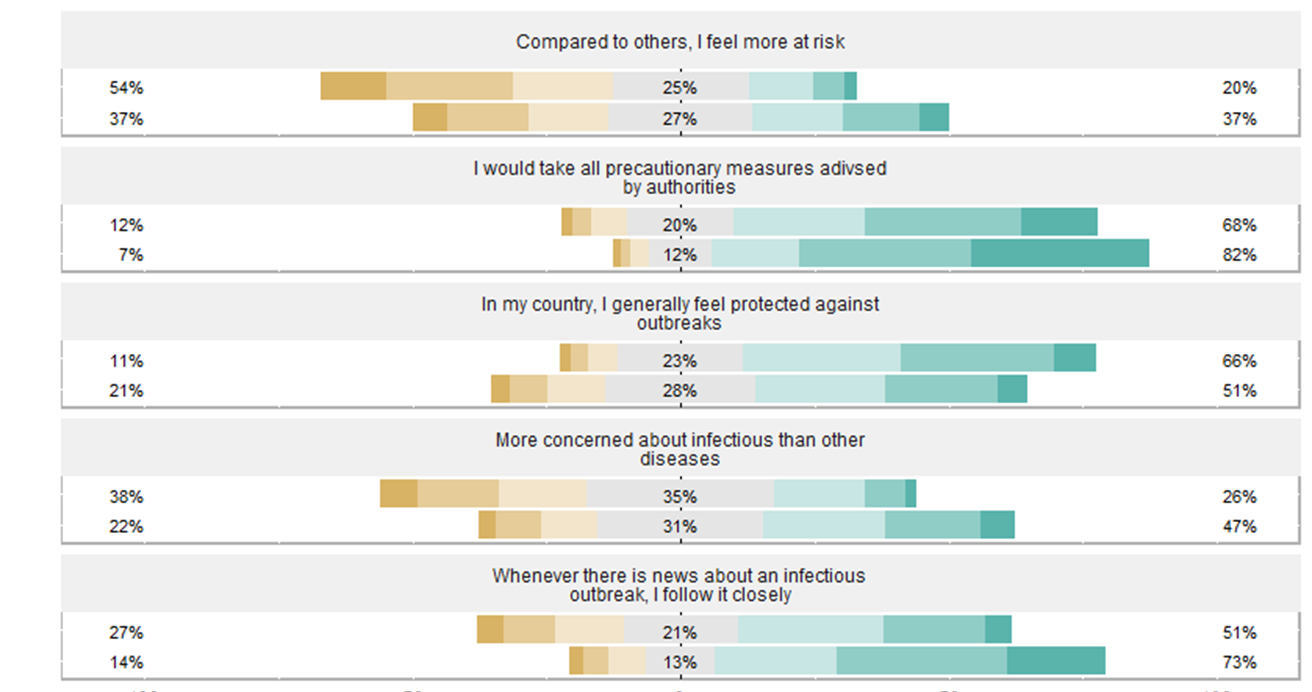

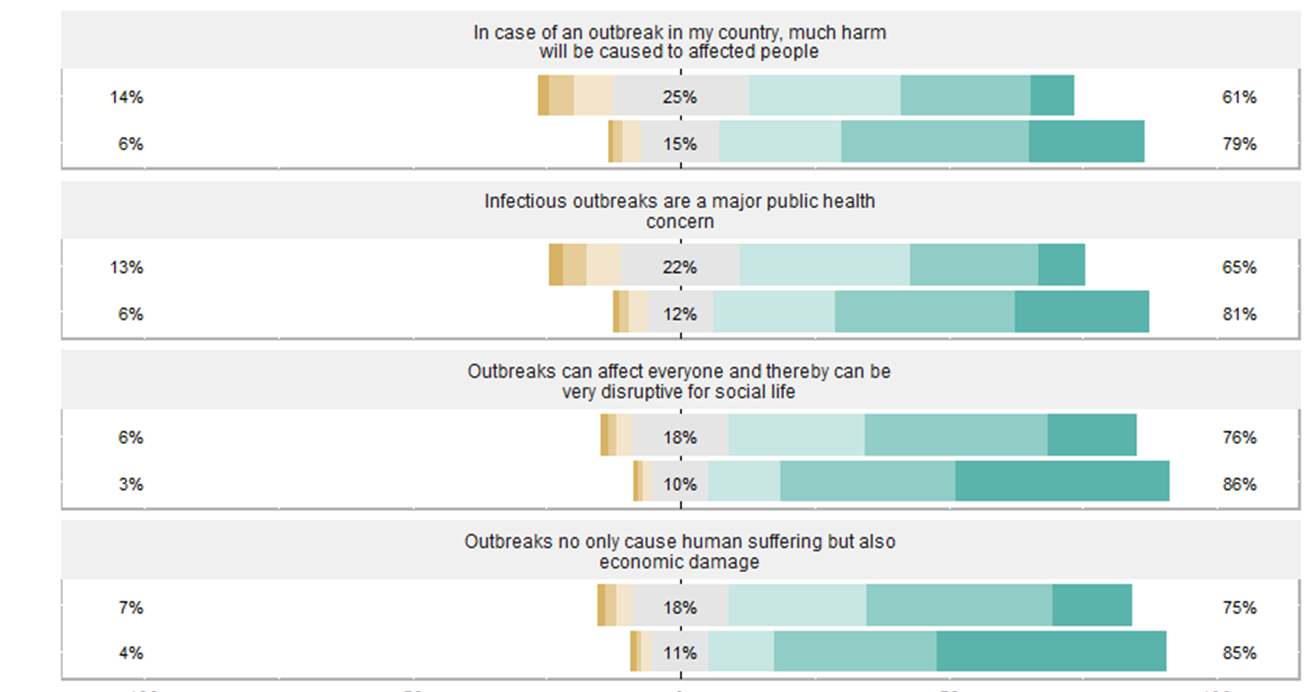

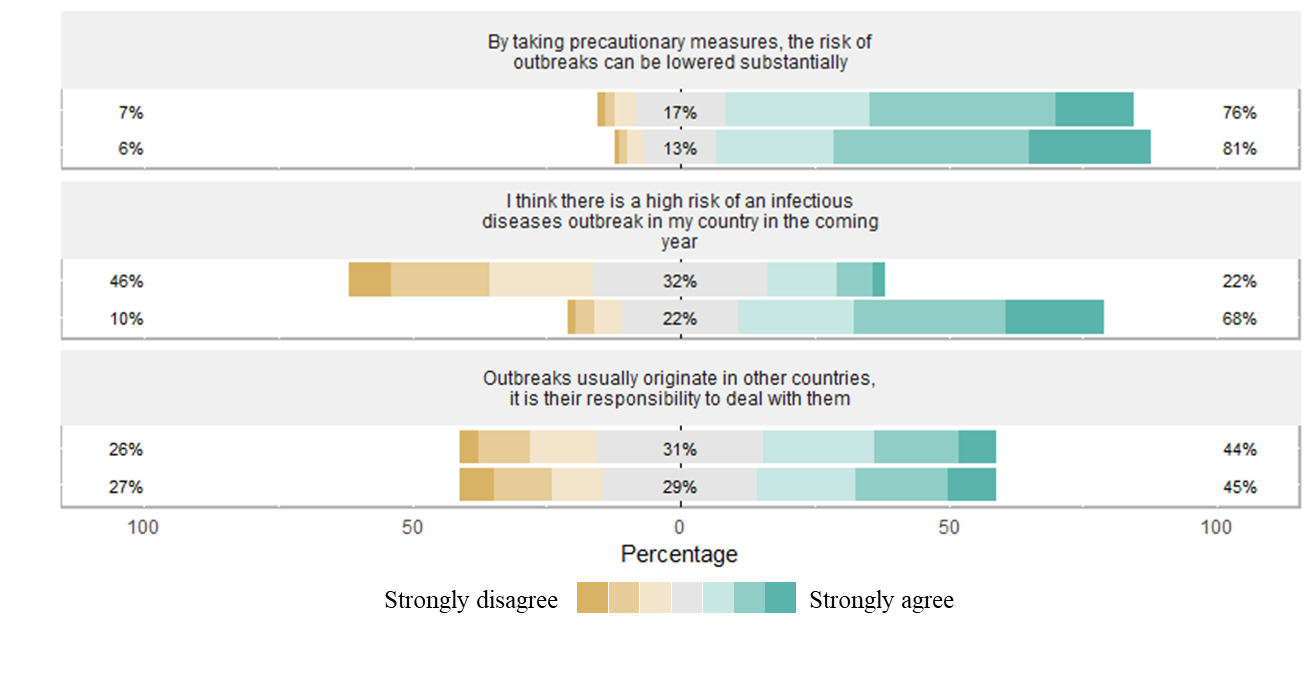
**

Figure A 1 Change in awareness of infectious disease outbreaks from 2018 (top) to 2020 (bottom). Weights were applied to account for the different country composition in 2020 (Italy double sample).


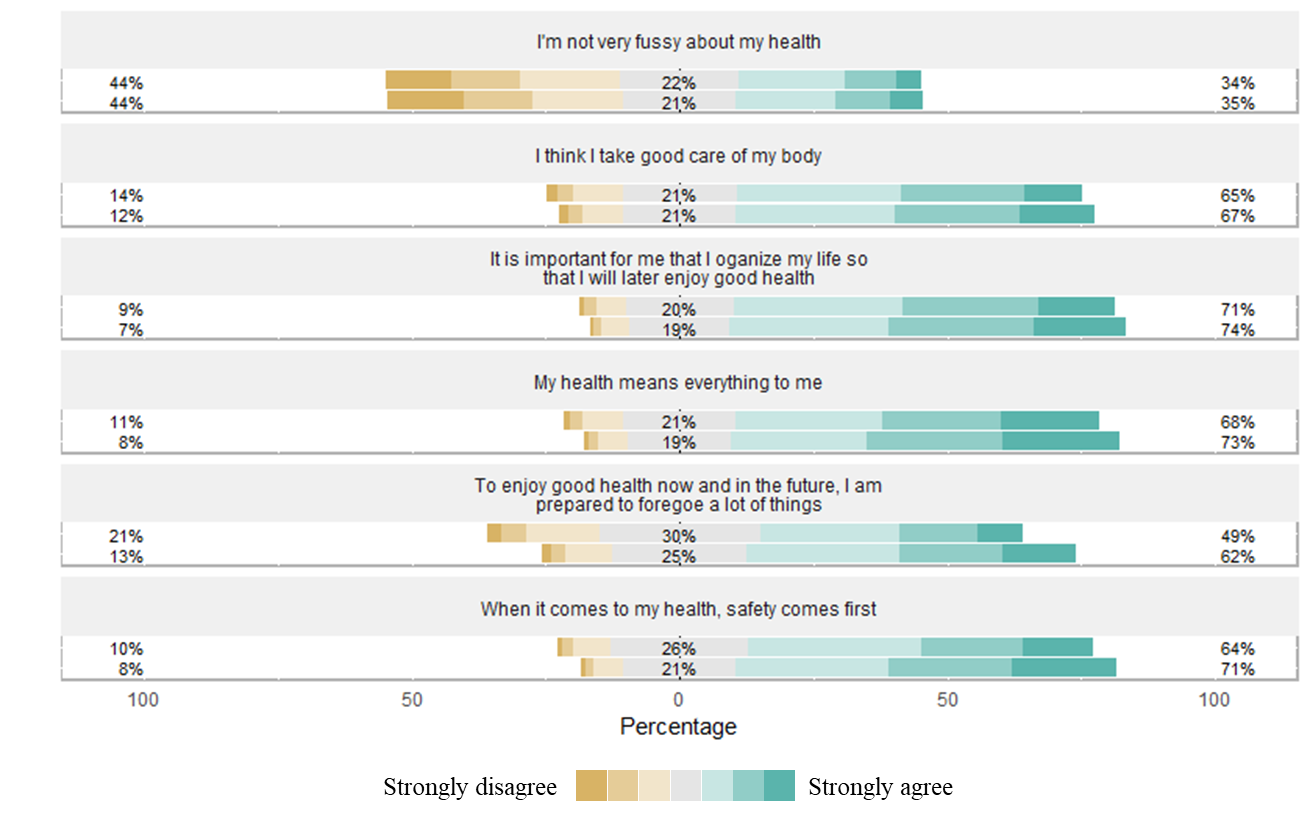


Figure A 2 Changes in health risk attitude from 2018 (top) to 2020 (bottom). Weights were applied to account for the different country composition in 2020 (Italy double sample).

Table A 4 Dataset conditioning and number of observations for different parts of the analysis.

|  | ‘System’ | |  | ‘Base case’ | |  | ‘Certainty’ | |  | ‘Death’ | |
| --- | --- | --- | --- | --- | --- | --- | --- | --- | --- | --- | --- |
|  | **2018** | **2020** |  | **2018** | **2020** |  | **2018** | **2020** |  | **2018** | **2020** |
|  |  |  |  |  |  |  |  |  |  |  |  |
| **Full observations** | 3,140 | 3,979 |  | 3,140 | 3,979 |  | 1,734 | 1,993 |  | 646 | 1,986 |
|  |  |  |  |  |  |  |  |  |  |  |  |
| - Outliers | 121 | 220 |  | 130 | 248 |  | 83 | 135 |  | 28 | 139 |
| - Protest answers | 306 | 290 |  | 289 | 261 |  | 170 | 152 |  | 40 | 135 |
|  |  |  |  |  |  |  |  |  |  |  |  |
| **WTP values analysed** | 2,713 | 3,469 |  | 2,721 | 3,470 |  | 1,481 | 1,706 |  | 578 | 1,712 |
|  |  |  |  |  |  |  |  |  |  |  |  |
| - Missing variable information^†^ | 327 | 323 |  | 332 | 325 |  | 185 | 166 |  | 69 | 157 |
|  |  |  |  |  |  |  |  |  |  |  |  |
| Included in regression analysis^¶^ | 2,386 | 3,146 |  | 2,389 | 3,145 |  | 1,296 | 1,540 |  | 509 | 1,555 |
|  |  |  |  |  |  |  |  |  |  |  |  |

*Note.* ^†^Predominantly missing income data.

Table A 5 Share of outliers, protest answers and zero responses.

|  | ‘System’ | | |  | ‘Base case’ | | | |  | ‘Certainty’ | | | |  | ‘Death’ | | |
| --- | --- | --- | --- | --- | --- | --- | --- | --- | --- | --- | --- | --- | --- | --- | --- | --- | --- |
|  | **2018** | **2020** | |  | **2018** | | **2020** | |  | **2018** | | **2020** | |  | **2018** | | **2020** |
| **Outliers** |  |  | |  |  | |  | |  |  | |  | |  |  | |  |
| DK | 0.012 | 0.034 | |  | 0.010 | | 0.034 | |  | 0.010 | | 0.017 | |  | 0.002 | | 0.028 |
| GER | 0.025 | 0.036 | |  | 0.025 | | 0.045 | |  | 0.023 | | 0.027 | |  | 0.004 | | 0.028 |
| HUN | 0.03 | 0.045 | |  | 0.052 | | 0.064 | |  | 0.024 | | 0.039 | |  | 0.01 | | 0.038 |
| IT | 0.08 | 0.106 | |  | 0.086 | | 0.104 | |  | 0.054 | | 0.057 | |  | 0.013 | | 0.056 |
| NL | 0.05 | 0.039 | |  | 0.04 | | 0.039 | |  | 0.021 | | 0.019 | |  | 0.01 | | 0.021 |
| UK | 0.034 | 0.03 | |  | 0.036 | | 0.052 | |  | 0.027 | | 0.025 | |  | 0.014 | | 0.022 |
| **Total** | **0.039** | **0.055** | |  | **0.041** | | **0.062** | |  | **0.026** | | **0.034** | |  | **0.009** | | **0.035** |
|  |  |  | |  |  | |  | |  |  | |  | |  |  | |  |
| **Protest answers** | |  | |  |  | |  | |  |  | |  | |  |  | |  |
| DK | 0.064 | 0.072 | |  | 0.084 | | 0.068 | |  | 0.054 | | 0.032 | |  | 0.012 | | 0.049 |
| GER | 0.086 | 0.121 | |  | 0.098 | | 0.096 | |  | 0.050 | | 0.058 | |  | 0.019 | | 0.042 |
| HUN | 0.167 | 0.073 | |  | 0.165 | | 0.071 | |  | 0.121 | | 0.039 | |  | 0.010 | | 0.045 |
| IT | 0.034 | 0.052 | |  | 0.033 | | 0.051 | |  | 0.025 | | 0.037 | |  | 0.010 | | 0.024 |
| NL | 0.103 | 0.087 | |  | 0.109 | | 0.087 | |  | 0.059 | | 0.048 | |  | 0.015 | | 0.039 |
| UK | 0.065 | 0.049 | |  | 0.069 | | 0.033 | |  | 0.020 | | 0.016 | |  | 0.011 | | 0.016 |
| **Total** | **0.086** | **0.073** | |  | **0.092** | | **0.066** | |  | **0.054** | | **0.038** | |  | **0.013** | | **0.034** |
|  |  |  | |  |  | |  | |  |  | |  | |  |  | |  |
| **Zero responses** | |  | |  |  | |  | |  |  | |  | |  |  | |  |
| DK | 0.054 | 0.053 | |  | 0.035 | | 0.055 | |  | 0.027 | | 0.027 | |  | 0.000 | | 0.017 |
| GER | 0.071 | 0.063 | |  | 0.038 | | 0.064 | |  | 0.031 | | 0.041 | |  | 0.006 | | 0.025 |
| HUN | 0.065 | 0.032 | |  | 0.046 | | 0.032 | |  | 0.048 | | 0.011 | |  | 0.004 | | 0.013 |
| IT | 0.038 | 0.025 | |  | 0.017 | | 0.025 | |  | 0.008 | | 0.011 | |  | 0.006 | | 0.009 |
| NL | 0.078 | 0.046 | |  | 0.059 | | 0.051 | |  | 0.036 | | 0.022 | |  | 0.011 | | 0.029 |
| UK | 0.063 | 0.038 | |  | 0.051 | | 0.038 | |  | 0.029 | | 0.025 | |  | 0.014 | | 0.016 |
| **Total** | **0.062** | **0.041** | |  | **0.041** | | **0.042** | |  | **0.030** | | **0.022** | |  | **0.007** | | **0.017** |
|  |  |  | |  |  | |  | |  |  | |  | |  |  | |  |
|  |  | -5%+ | -5% | | | -3% | | +/- 1% | | | +3% | | +5% | | | 5%+ |  |
|  |  |  | |  |  | |  | |  |  | |  | |  |  | |  |

*Note.* Outliers defined as WTP larger than 5% of monthly income. Zero responses after outliers and protest answers were excluded.


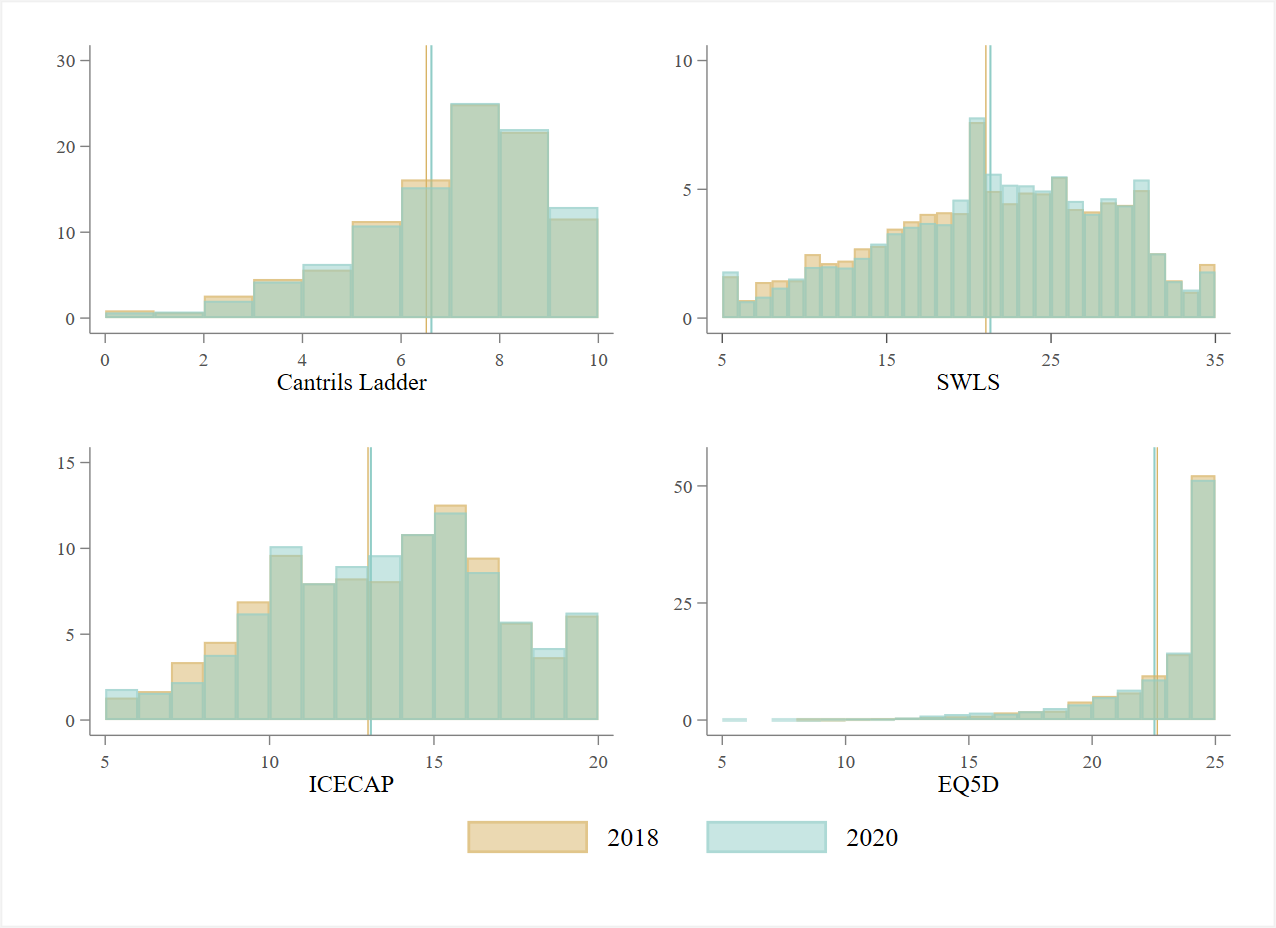


Figure A 3 Well-being and health across timepoints.
*Note.* SWLS, ICECAP and EQ5D calculated as unweighted sum scores. Percentages shown. Horizontal lines represent means.

Table A 6 Determinants of willingness to pay – Repeated sample

|  | **2018** |  | **2020** |  | ***P* Difference** |
| --- | --- | --- | --- | --- | --- |
| **Socio-economics status** |  |  |  |  |  |
| Log income | 9.59^***^ | (2.02) | 24.60^**^ | (12.24) | 0.183 |
| Age (Δ5 years) | -1.06 | (2.92) | -3.50 | (3.39) | 0.498 |
| Age-squared | -0.02 | (0.17) | 0.14 | (0.18) | 0.430 |
| Female | -2.05 | (2.15) | 2.68 | (4.41) | 0.229 |
| Tertiary education | 1.23 | (2.21) | -0.43 | (2.24) | 0.564 |
| Married | 1.54 | (1.88) | -11.52 | (9.99) | 0.193 |
| Self-employed | 1.82 | (3.83) | 1.32 | (4.05) | 0.930 |
| Not employed | -1.48 | (2.04) | 2.85 | (4.93) | 0.359 |
| EQ-5D-5L sum score (Δ5 points) | -0.23 | (0.46) | 0.06 | (0.45) | 0.614 |
|  |  |  |  |  |  |
| **Awareness of outbreaks** |  |  |  |  |  |
| Personal risk perception (Δ5 points) | 8.29^***^ | (1.95) | 6.86^***^ | (2.28) | 0.445 |
| Societal consequences (Δ5 points) | -1.44 | (1.57) | -5.87^***^ | (1.57) | 0.042 |
| Risk and response (Δ5 points) | -4.90 | (3.10) | -2.57 | (3.08) | 0.516 |
|  |  |  |  |  |  |
| **Past exposure** | 0.03 | (2.37) | 1.41 | (2.31) | 0.650 |
|  |  |  |  |  |  |
| **Health risk attitude** |  |  |  |  |  |
| HRAS-SF Q2 | 5.27^**^ | (2.65) | 5.35^*^ | (2.87) | 0.983 |
| HRAS-SF Q3 | -1.91 | (2.37) | 3.73 | (2.95) | 0.101 |
| HRAS-SF Q4 | 3.47 | (3.22) | 4.03 | (2.76) | 0.880 |
| Observations | 1,384 |  | 1,384 |  |  |
| Adjusted R-squared | 0.176 |  | 0.209 |  |  |
| Chow test statistics | 19.21 | *P* = 0.57 |  |  |  |

*Note.* WTP values from all four scenarios as dependent variable. Standard errors were clustered on individual level and are presented in parentheses. Northern Italy subsample from 2020 excluded. Country dummies and constant omitted from table. Regression is weighted by 2018 country sample sizes. ^*^ *p* < 0.10, ^**^ *p* < 0.05, ^***^ *p* < 0.01.

Table A 7 Determinants of willingness to pay – Italy

|  | **2018** |  | **2020** |  | ***P* Difference** |
| --- | --- | --- | --- | --- | --- |
| **Socio-economics status** |  |  |  |  |  |
| Log income | 11.53^***^ | (3.10) | 19.23^***^ | (2.63) | 0.059 |
| Age (Δ5 years) | -7.15^*^ | (4.01) | -8.22^**^ | (3.81) | 0.846 |
| Age-squared | 0.33 | (0.24) | 0.38^*^ | (0.22) | 0.876 |
| Female | 1.82 | (3.19) | 5.70^*^ | (3.24) | 0.392 |
| Tertiary education | -0.68 | (3.45) | 0.66 | (3.24) | 0.775 |
| Married | 4.97 | (3.13) | 0.44 | (2.80) | 0.277 |
| Self-employed | 4.63 | (4.56) | 6.41 | (4.10) | 0.773 |
| Not employed | -5.82^*^ | (3.43) | -3.50 | (3.18) | 0.617 |
| EQ-5D-5L sum score (Δ5 points) | -2.37^***^ | (0.74) | -0.81 | (0.58) | 0.094 |
|  |  |  |  |  |  |
| **Awareness of outbreaks** |  |  |  |  |  |
| Personal risk perception (Δ5 points) | 4.97^*^ | (2.67) | 6.62^***^ | (2.27) | 0.635 |
| Societal consequences (Δ5 points) | 1.67 | (2.72) | -7.64^**^ | (3.12) | 0.024 |
| Risk and response (Δ5 points) | -3.09 | (3.73) | 4.31 | (3.07) | 0.125 |
|  |  |  |  |  |  |
| **Past exposure** | 21.37^***^ | (5.72) | 6.01 | (3.66) | 0.021 |
|  |  |  |  |  |  |
| **Health risk attitude** |  |  |  |  |  |
| HRAS-SF Q2 | -2.44 | (4.63) | -3.39 | (4.28) | 0.883 |
| HRAS-SF Q3 | -7.03 | (4.42) | -5.09 | (4.04) | 0.745 |
| HRAS-SF Q4 | 0.30 | (4.60) | 3.43 | (3.96) | 0.604 |
| Observations | 1,120 |  | 1,498 |  |  |
| Adjusted R-squared | 0.221 |  | 0.211 |  |  |
| Chow test statistics | 22.53 | *P =* 0.13 |  |  |  |

*Note.* WTP values from all four scenarios as dependent variable. Standard errors were clustered on individual level and are presented in parentheses. Northern Italy subsample from 2020 excluded. Country dummies and constant omitted from table. Regression is weighted by 2018 country sample sizes. ^*^ *p* < 0.10, ^**^ *p* < 0.05, ^***^ *p* < 0.01.

******

Figure A 4 Distribution of differences between WTP values of 2018 and 2020 for individuals sampled twice.
*Note.* WTP values trimmed at +/- 100€. Red line represents the mean difference.

Table A 8 Calculation of aggregate WTP or ‘maximum willingness to be taxed’

| Country | Median monthly WTP (€)^1^  **2018** | Median monthly WTP (€)^1^  **2020** | Protest zero^3^  **2018** | Protest zero^3^  **2020** | # households in million | HH paying tax^2^ | Aggregate per year (bn €)  **2018** | Aggregate per year (bn €)  **2020** |
| --- | --- | --- | --- | --- | --- | --- | --- | --- |
| UK | 8.6 | 12.2 | 6.8% | 4.9% | 27.2m | 50% | **1.31** | **1.89** |
| Denmark | 10.4 | 14.8 | 6.8% | 7.2% | 2.69m | 50% | **0.16** | **0.22** |
| Germany | 8.1 | 9.5 | 10.0% | 12.1% | 41.31m | 50% | **1.83** | **2.07** |
| Hungary | 5.4 | 7.6 | 18.2% | 7.3% | 4.22m | 50% | **0.11** | **0.18** |
| Italy | 15.5 | 20.4 | 4.6% | 5.2% | 25.6m | 50% | **2.30** | **2.97** |
| Netherlands | 7.7 | 11.1 | 12.4% | 8.7% | 7.79m | 50% | **0.32** | **0.47** |
| **Total** |  |  |  |  |  |  | **6.04** | **7.81** |

^1^ Based on data presented in Figure 3

^2^ Assumption based on the share of households with income taxpayer who are eligible for additional taxation.

^3^ Based on Table A 5
